# Supplementary material for: Endoscopic ultrasound-guided radiofrequency ablation (EUS-RFA) for advanced pancreatic and periampullary adenocarcinoma
Source: Sci Rep. 2022 Oct 3;12:16516. doi: 10.1038/s41598-022-20316-2 (PMC9530230; doi:10.1038/s41598-022-20316-2)
Supplement: Supplementary file 1 — Supplementary Information 1. [file 41598_2022_20316_MOESM1_ESM.pdf]

PID005	Date	value	week	PID006	date	value
	11/14/2016	5,941.70	6		**1/16/2017**	**3,989.90**
	11/7/2016	4,946.50	5		12/22/2016	673.4
	10/31/2016	3,721.20	**4**		11/29/2016	413.3
	**10/24/2016**	**2,875.90**	3		11/9/2016	464.9
	10/10/2016	2,439.00	1		10/28/2016	480.1
	9/12/2016	2,231.10			10/18/2016	584.7
	9/7/2016	2,031.40			10/7/2016	539.4
	8/29/2016	1,591.90			9/23/2016	541.6
	8/18/2016	1,289.00			9/9/2016	490.1
	8/10/2016	428.3			8/15/2016	442.2
	7/28/2016	696.2			8/5/2016	453.6
	7/14/2016	451			7/26/2016	600.3
	6/1/2016	164.4			7/15/2016	587.5
	5/11/2016	273.4			7/5/2016	687.3
	4/11/2016	605.3			6/24/2016	1,111.50
	**2/4/2016**	**938.3**			6/10/2016	1,013.10
					5/31/2016	1,078.60
					5/23/2016	1,188.00
					5/10/2016	1,408.80
					4/29/2016	1,484.20
					**4/19/2016**	**1,669.10**

week	PID008	date	value	week	PID009	11/12/2020
1		7/19/2017	2,709.30	27		10/29/2020
		6/21/2017	498.6	23		10/15/2020
		6/7/2017	351.3	21		10/1/2020
		5/10/2017	417	17		9/17/2020
		4/12/2017	485.9	13		9/3/2020
		3/29/2017	425	11		8/20/2020
		3/15/2017	622.4	9		7/23/2020
		3/2/2017	642.8	7		**7/9/2020**
		2/23/2017	519.1	**6**		6/25/2020
		**2/16/2017**	**407.4**	5		6/11/2020
		2/2/2017	388.3	3		5/28/2020
		1/11/2017	119	**1**		5/14/2020
		**1/4/2017**	**135.7**			4/30/2020
		12/21/2016	202.9			4/16/2020
		12/14/2016	161			4/2/2020
						3/5/2020
						2/20/2020
						2/6/2020
						1/9/2020
						12/26/2019
						12/12/2019
						**11/29/2019**

27	PID011	date	value	week	PID012
26		10/19/2017	7000	26	
26		9/28/2017	5,602.50	23	
27		9/8/2017	1,715.90	20	
		8/16/2017	1,765.60	17	
16		8/2/2017	2,022.70	15	
		7/19/2017	2,513.90	13	
30.2		6/21/2017	1,629.40	9	
**23.5**		6/7/2017	1,849.50	**7**	
		**5/24/2017**	**1,022.00**	5	
		5/10/2017	1,567.30	3	
		4/26/2017	2,316.40	1	
		4/12/2017	5,340.00		
		3/29/2017	1,969.70		
		3/16/2017	2,516.80		
		3/1/2017	696		
		2/15/2017	204.1		
		**2/8/2017**	**230.5**		
		1/25/2017	162		
		1/18/2017	204.6		
13		1/11/2017	249.7		
20.8		12/28/2016	326.9		
**8.1**		12/14/2016	186.6		
		11/30/2016	213.9		
		11/23/2016	207.5		
		11/2/2016	190.9		
		10/26/2016 0:00	177.5		
		9/28/2016	247.1		
		9/21/2016 0:00	265		
		9/14/2016 0:00	303		
		9/7/2016 0:00	357.3		
		8/24/2016 0:00	476.9		
		8/17/2016 0:00	543.7		
		7/18/2016 0:00	1309		
		7/7/2016 0:00	1773.2		

date	value	week	PID013	date	value	week
9/3/2019	47.3	115		2/15/2018	56.4	38
8/29/2019	45.2	114		1/8/2018	33.1	35
8/15/2019	77.8	112		1/8/2018	30.3	33
8/6/2019	46.6	111		12/11/2017	25	30
8/1/2019	143.8	110		11/29/2017	22.8	27
7/18/2019	53	108		11/1/2017	21	23
7/11/2019	170.8	107		10/4/2017	17.8	19
6/28/2019	117.1	105		9/28/2017	15.9	**18**
6/13/2019	75	**103**		**9/13/2017**	**16.9**	16
**5/30/2019**	**67.1**	101		9/6/2017	28.4	15
5/13/2019	53.6	99		8/23/2017	20.5	13
5/10/2019	33.9	**98**		8/16/2017	22.4	12
**5/2/2019**	**46.2 H**	97		8/9/2017	28.5	11
4/23/2019	74.7	95		7/26/2017	26.3	10
4//11/2019	69.2	93		7/12/2017	37.1	8
4/4/2019	170.3	92		5/31/2017	52.9	**2**
3/21/2019	141	90		**5/24/2017**	**52.5**	1
3/14/2019	153	89				
2/27/2019	177.8	87				
2/21/2019	198	86				
2/7/2019	169	84				
1/31/2019	238	83				
1/17/2019	297.5	81				
1/10/2019	273.8	80				
12/27/2018	549.9	78				
12/13/2018	492.2	76				
11/29/2018	286.5	74				
11/21/2018	853	73				
11/15/2018	841	72				
11/7/2018	603.6	71				
9/13/2018	122.5	66				
8/2/2018	82.1	63				
6/20/2018	24	57				
6/4/2018	13	55				
5/3/2018	11.8	51				
4/19/2018	14.4	49				
4/5/2018	21.8	47				
3/21/2018	23.1	45				
3/8/2018	33.1	43				
2/22/2018	23.6	41				
11/22/2017	11.9	29				
11/9/2017	16.1	27				
10/30/2017	15.7	25				
10/12/2017	15.9	23				
9/14/2017	9.7	19				

8/24/2017	16.9	16
8/10/2017	23.1	14
8/3/2017	38.1	13
7/13/2017	18.5	10
7/6/2017	22.9	9
6/22/2017	34	7
5/25/2017	62.4	3
4/17/2017	22.4	1
12/12/2016	6.7	
9/26/2016	12.8	
8/15/2016	11.5	
6/27/2016	15.5	
5/2/2016	27.2	
4/11/2016	10.7	
3/29/2016	22.9	

PID021	date	value	week
	8/3/2020	136	157
	6/18/2020	150	151
	5/6/2020	151	146
	3/19/2020	126.8	139
	3/2/2020	133.6	**137**
	**1/24/2020**	**399.9**	132
	10/7/2019	60	117
	9/11/2019	67.9	113
	8/13/2019	64.8	109
	7/23/2019	51	**106**
	**7/3/2019**	**47.6**	103
	6/18/2019	68.2	101
	6/6/2019	53.2	99
	5/8/2019	31.7	95
	4/6/2019	70	91
	2/21/2019	66.8	84
	1/24/2019	66.6	80
	8/1/2018	55.6	57
	6/11/2018	50.1	51
	5/29/2018	58.8	49
	5/23/2018	67.8	48
	5/16/2018	67	47
	5/2/2018	169	45
	4/17/2018	106.2	43
	4/3/2018	86.9	41
	3/21/2018	263.9	39
	3/8/2018	528.9	37
	2/15/2018	92.6	34
	2/1/2018	82.6	32
	1/18/2018	62.1	30
	1/4/2018	87.5	28
	12/28/2107	75.8	27
	12/14/2017	71	25
	11/27/2017	122.9	23
	11/9/2017	107.7	20
	10/26/2017	92.4	18
	10/12/2017	82.5	16
	9/28/2017	125.8	14
	9/14/2017	176.6	12
	8/31/3017	149.8	10
	8/17/2017	203.3	8
	8/3/2017	214.7	6
	7/20/2017	313.5	4
	7/10/2017	687.5	3
	7/7/2017	414.4	2

**PID003**	12/14/2017	44
	11/30/2017	43.3
	**10/26/2017**	**30.8**
	10/12/2017	36
	9/28/2017	39
	9/21/2017	37.8
	9/14/2017	38
	8/14/2017	
	7/31/2017	31.8
	7/3/2017	27.8
	6/19/2017	25.4
	5/31/2017	20.2
	5/22/2017	
	**5/8/2017**	**25.2**

6/30/2017	248.3	1

PID010	6/4/2018	114.5	64
	5/29/2018	144	63
	**5/17/2018**	**183.7**	61
	5/11/2018	196.1	60
	4/16/2018	164	56
	4/9/2018	411.8	55
	3/26/2018	179.3	54
	3/19/2018	184.2	53
	3/12/2018	199.5	52
	2/26/2018	153	50
	2/19/2018	118.4	49
	2/12/2018	116.2	48
	2/8/2018	108.1	47
	**1/31/2018**	**105.4**	46
	1/25/2018	126.7	45
	12/13/2017	111.4	40
	11/29/2017	93.2	38
	11/22/2017	111.2	37
	11/15/2017	113.7	36
	11/1/2017	76.5	34
	10/25/2017	71.7	25
	10/18/2017	72.8	23
	10/4/2017	64.8	21
	9/27/2017	65.5	20
	9/20/2017	65.8	19
	9/6/2017	96.4	17
	8/23/2017	72.3	15
	8/9/2017	153.5	13
	8/3/2017	1242.4	12
	7/26/2017	83.5	11
	7/12/2017	93.9	9
	7/5/2017	97.8	8
	6/28/2017	110.8	7
	6/14/2017	144.8	5
	5/31/2017	114.4	3
	5/17/2017	250.6	1
	5/9/2017	142	
	5/3/2017	209.6	
	4/19/2017	154.9	
	4/12/2017	158.4	
	4/5/2017	183	
	3/29/2017	309	
	3/24/2017	636	
	3/15/2017	1696	
	3/8/2017	554.8	
	2/22/2017	435	

2/1/2017	432
1/26/2017	281
