# Supplementary material for: Endoscopic ultrasound-guided radiofrequency ablation (EUS-RFA) for advanced pancreatic and periampullary adenocarcinoma
Source: Sci Rep. 2022 Oct 3;12:16516. doi: 10.1038/s41598-022-20316-2 (PMC9530230; doi:10.1038/s41598-022-20316-2)
Supplement: Supplementary file 2 — Supplementary Information 2. [file 41598_2022_20316_MOESM2_ESM.pdf]

StudyID	Age	Gender	Race	Location of Lesion	Diagnosis
PID005	62	Female	White/Caucasian	Head	Panc AdenoCa-Stage 3
PID006	55	Male	White/Caucasian	Body	Panc AdenoCa-Stage 4
PID008	73	Male	White/Caucasian	Head	Panc AdenoCa-Stage 3
PID009	59	Male	White/Caucasian	Head	Panc AdenoCa-Stage 4
PID011	66	Male	Asian	Tail	Panc AdenoCa-Stage 4
PID012	68	Male	White/Caucasian	Tail	Panc AdenoCa-Stage 3
PID013	59	Female	White/Caucasian	Neck	Panc AdenoCa-Stage 3
PID021	57	Male	Asian	Head	Panc AdenoCa-Stage 3
**PID003**	**62**	**Male**	**White/Caucasian**	**Neck**	**Panc AdenoCa-Stage 3**
PID010	62	Female	White/Caucasian	Body	Panc AdenoCa-Stage 3

Pre Treatment Size	Post Treatment Size	Symptoms Related To Lesion	TechnicalSuccess	Describe Clinical Outcome	Procedure Related Complications
30 x 30	NA	Yes	Yes		Yes
32 x 19	NA	No	Yes		No
42 x 31	44 x 43	No	Yes		No
14 x 12	0x0	No	Yes		No
29 x 44	17 x 25	No	Yes		No
15 x 10	12 x 7	No	Yes		No
38 x 38	50 x 40	No	Yes		No
25 x 22	10x 21	No	Yes		No
**23 x 24**	**15 x 19**	**No**	**Yes**	**improvement in lesion size**	**No**
22 x 18	18 x 16	No	Yes		No

Date of Diagnosis	Chemotherapy	Date of Diagnosis	Size at Diagnosis on Imaging (mm)	Size at Diagnosis on EUS (mm)
2/3/16	FOLFIRINOX, GemAbraxane	2/3/16	20	30 x 20
10/21/14	FOLFIRINOX, GemAbraxane	10/21/14	50 x 47 x 68	60
10/4/16	FOLFIRINOX, GemAbraxane	10/4/16	NA	40 x 33
10/1/15	Xeloda, FOLFOX, FOLFIRINOX	10/1/15	19	14 x 12
6/22/16	FOLFIRINOX	6/22/16	52 x 40	53 x 39
3/10/16	GemAbraxane	3/10/16	26	21 x 18
5/23/17	FOLFIRINOX	5/23/17		35 x 35
6/18/17	FOLFIRINOX, Capecitabine	6/18/17	27 x 19 x 30	28x25
**2/25/16**	FOLFIRINOX, GemAbraxane	2/25/16	50	59x47
1/25/17	FOLFIRINOX, GemAbraxane	1/25/17	47 x 44 x 42	44x40

Total RFA Sessions	Date of Index RFA	Pre Treatment Size on Index EUS RFA	Date of Death	Date of Last Follow Up	Survival in Months
1	10/26/2016	30 x 30	12/11/16		11.25
1	10/21/2014	32 x 50	2/1/17		29.75
2	10/4/2016	42 x 31	8/2/17		10.75
2	10/1/2015	14 x 12	ALIVE	4/28/2022	78.90
1	6/22/2016	23 x 27	12/1/17		18.75
4	3/10/2016	15 x 10	9/16/19		46
3	5/23/2017	38 x 34	2/8/19		10
3	6/18/2017	24 x 21	ALIVE	5/9/2022	58.7
1	2/25/2016	23 x20	2/2/18		25.25
4	1/25/2017	44x22	6/22/18		18.25
